# Lithium, trifluperazine and idiopathic leucopenia: Author and reviewer perspectives on how to write a good case report

**DOI:** 10.4103/0019-5545.64594

**Published:** 2010

**Authors:** Chittaranjan Andrade, Dattatreya N Mendhekar

**Affiliations:** Department of Psychopharmacology, National Institute of Mental Health and Neurosciences, Bangalore, India; 1 Neuropsychiatry and Headache Clinic, Prathap Nagar Metro Station, New Delhi, India

**Keywords:** Case report preparation, idiopathic leucopenia, lithium, surviving peer review, trifluperazine, writing skills

## Abstract

**Background:**

The Indian *Journal of Psychiatry* receives many reports which, despite obvious academic worth, are too poorly written to be publishable. Such submissions tax manuscript reviewers and increase the editorial office workload without benefiting the authors with a publication.

**Methods:**

We describe an authentic and previously unpublished case of idiopathic leucopenia and psychosis. Leucocyte levels in this patient dropped upon challenge with different atypical antipsychotic drugs. Lithium pretreatment, however, permitted the safe and successful use of trifluperazine. Readers are invited to use a roughly-prepared version of the case report to draft a submission-worthy manuscript.

**Results:**

Two versions of the manuscript are presented. The first version is generally satisfactory but will trigger several queries during peer review; these queries are indicated. The second version would be considered acceptable by most reviewers.

**Conclusions:**

Readers who work through the exercise provided in this article will better understand how authors should prepare their report and how reviewers may scrutinize their manuscript.

## INTRODUCTION

The *Indian Journal of Psychiatry* receives far more manuscripts than it can publish. Regrettably, although many of these manuscripts are academically worthy, they are too poorly written to merit acceptance. Some manuscripts are considered beyond salvage; these are, regrettably, rejected outright. Other manuscripts pass through one or more rounds of peer review before an editorial decision is taken. In both situations, and especially in the latter, the editorial office and the reviewers of the manuscripts are taxed; this wastes time and resources, and the waste is a complete loss for all involved if the manuscript is eventually rejected.

This article therefore seeks to help authors understand how to prepare a reasonably well-written manuscript using a case report by way of example. The objectives of this article are to improve the chances that authors will receive a favorable review for their own manuscripts, and to thereby reduce the burden that the reviewers and editorial office of the *Indian Journal of Psychiatry* experience with submissions that require extensive and repeated rounds of revision.

## METHODS

In this section, we present an authentic and previously unpublished case which illustrates the use of lithium to prevent an adverse hematological reaction in a young woman with childhood-onset idiopathic leucopenia and recent-onset psychosis. As an exercise in manuscript-preparation skills, we invite readers to use the information in the summary presented below to draft a case report which can be submitted to a journal for consideration for publication. The summary has deliberately been prepared as a roughly-written draft in order to mimic an unsophisticated attempt at manuscript preparation.

In the next (Results) section, we present a version of the case report which is generally satisfactory but which would elicit several enquiries, comments, and suggestions for improvement during the peer review process. We briefly indicate concerns that the reviewers may express. Finally, we present a version of the report which would likely be considered acceptable by most reviewers. Readers may note that the references for the different versions are presented not in the text but at the end of this article.

## ROUGH DRAFT

Psychosis treated successfully with trifluperazine in a case of idiopathic blood dyscrasia by pre-treatment with lithium

Literature is silent on the management of psychosis in a known case of preexisting blood dyscrasia. Thus we report a young girl with leucopenia who developed psychosis but was found to be hematologically sensitive to atypical antipsychotic drugs. She was started put on lithium to prevent further decline in blood count and then successfully treated with trifluperazine.

### Case report

SK, a 23 years old girl was a known case of idiopathic leucopenia since her childhood. Her total WBC count was always <3500/mm^3^ (normal, 4,300 to 10,800). However her differential counts were within normal limits including neutrophil and platelet count. Even her hemoglobin count was always 11-13 gm%.

She was investigated extensively but the specific cause for blood dyscrasia was not found. Hence she was diagnosed as idiopathic leukopenia after consulting hematologists. Parental granulocyte colony stimulating factors could raise the blood count only to lower side of normal range (4000-4600/mm^3^ ). In 2005 she had first psychiatric consultation with the history of extremely withdrawn behavior, negativism, poor personal care and muttering to self. In view of psychotic nature of illness, she was treated unsuccessfully with separate trials of risperidone, ziprasidone, aripiprazole and quetiapine. All these drugs were discontinued within 10-15 days of administration as her blood count fell further (WBC count 1600-2500/mm^3^ ). However subsequently she showed partial remission in her psychotic illness. After 6 months she had relapse into similar psychotic symptoms and that time baseline WBC count was 2900/mm^3^ . In view of past history of hematological sensitivity to antipsychotic drugs lithium 300 mg/day was started. Two weeks later trifluperazine 5 mg was added to lithium therapy which was next day increased to 10 mg/day. At day 7 of combined therapy there was no change in blood count. At the end of 4 weeks marked improvement was noticed in psychotic illness. This regime was continued for 8 weeks and tapered and stopped without any marked changes in blood count. In 2008 also her relapse into psychosis was treated in a similar fashion without any adverse events. As the case was not cooperative for drawing blood even during remission phase we could not get enough blood samples for serum lithium test.

### Discussion

This report showed that no atypical antipsychotic drug is safe in a patient with psychosis suffering from blood dyscrasia. Although here lithium is not indicated but it was administered to increase leucocyte count. Lithium has been reported to ameliorate neutropenia associated with clozapine and olanzapine therapies.[1,2] Lithium has also been shown to cause leucocytosis by stimulating granulocyte colony growth factor. This study provides support for the utility for lithium in further decline of WBC count when combined with typical neuroleptic, however, extra vigilance may be required.

## RESULTS

After reorganization, rewriting, and addition of additional necessary information, the case report may appear as in Version 1, presented below in a form which can be submitted as a brief report without an abstract or as a letter to the editor. Readers are invited to observe changes made to the title, organization, grammar, and manner of expression of content. Readers are also invited to note contents that have been added or as deleted. Capitalized in parentheses are concerns that reviewers may express and clarifications that they may seek. Version 2, with an improved title, is what most reviewers and editors might find acceptable for publication. We remind readers that the references for the two versions are presented at the end of this article.

## VERSION 1

Lithium pretreatment permits trifluperazine use in a case of idiopathic leucopenia and psychosis

Lithium stimulates leucocytosis by inducing granulocyte colony stimulating factor (GCSF).[3,4] Lithium [SPECIFY DOSE RANGE] has been used with success to ameliorate neutropenia associated with olanzapine[1] and clozapine[2] treatment. We describe a patient with childhood-onset idiopathic leucopenia who developed psychosis during adult life, who was hematologically sensitive to several antipsychotic drugs, and who was eventually successfully treated with trifluperazine (TFP) after pretreatment with lithium.

### Case report

SK, a 23-year-old woman, was a known case of childhood-onset idiopathic leucopenia. Her total leucocyte (WBC) count was always below 3500/mm^3^ with differential count, platelet count, and hemoglobin level within normal limits. GCSF treatment could raise the blood count only to the lower side of the normal range (4000-4600/mm^3^ ).

During 2005, she was brought into psychiatric care for the management of social withdrawal, poor personal care, negativism, and talking-to-self behavior. She fulfilled DSM-IV criteria for undifferentiated schizophrenia and was prescribed antipsychotic medication. However, trials of risperidone, ziprasidone, aripiprazole, and quetiapine were each aborted within 10-15 days of commencement because her WBC count dropped into the 1600-2500/mm^3^ range. After 6 months of unsuccessful management without medications, she was started on lithium (300 mg/day) in the hope that this drug would stimulate leucocytosis and permit successful antipsychotic initiation.

Her pre-lithium WBC count was 2900/mm^3^; this rose [PLEASE PROVIDE VALUE] after two weeks of lithium therapy, at which point TFP was added. The TFP dose was 5 mg on the first day and 10 mg/day thereafter. By the end of a month of lithium-TFP combination therapy, psychosis was markedly attenuated. Treatment was continued for a further month and then tapered and withdrawn [WHY WASN’T TREATMENT CONTINUED INDEFINITELY?]. The WBC count remained stable [SPECIFY RANGE] during the entire period of combination therapy.

She experienced a relapse of psychosis during 2008 and was successfully and uneventfully treated with the lithium-TFP combination as before [PLEASE PROVIDE MORE DETAILS].

### Discussion

GCSF [REFERENCE][4] and lithium[5–9] have both been used to treat blood dyscrasias that arise during antipsychotic therapy. There is, however, no literature on the treatment of psychosis in patients with preexisting idiopathic blood dyscrasias that are worsened by antipsychotic drugs. One possible interpretation of the case that we describe is that TFP had no adverse effect on WBC levels. However, considering that four previous antipsychotic drugs had each worsened the dyscrasia, we suggest an alternate explanation: that that low-dose lithium pretreatment may help some patients with idiopathic blood dyscrasias (that are sensitive to psychotropic drugs) to successfully receive psychotropic medication. The successful use of lithium [SPECIFY DOSE] to prevent clozapine-induced neutropenia[5] supports this view.

[THIS PAPER DOES NOT REFERENCE SEVERAL OTHER RELEVANT REPORTS AND REVIEWS.]

## VERSION 2: THE FINAL VERSION

Lithium pretreatment permits trifluperazine use in a psychotic woman with antipsychotic drug-sensitive idiopathic leucopenia

Lithium stimulates leucocytosis by inducing granulocyte colony stimulating factor (1) (GCSF). Lithium (300-900 mg/day) has been used with success to correct neutropenia associated with olanzapine (2) and clozapine (3-7) treatment. We describe a patient with childhood-onset idiopathic leucopenia who developed psychosis during adult life, who was hematologically sensitive to several antipsychotic drugs, and who was eventually successfully treated with trifluperazine (TFP) after pretreatment with lithium.

### Case report

SK, a 23-year-old woman, was a known case of childhood-onset idiopathic leucopenia. Her total leucocyte (WBC) count was always below 3500/mm^3^ with differential count, platelet count, and hemoglobin level within normal limits. GCSF treatment could raise the blood count only to the lower side of the normal range (4000-4600/mm^3^ ).

During 2005, she was brought into psychiatric care for the management of social withdrawal, poor personal care, negativism, and talking-to-self behavior. She fulfilled DSM-IV criteria for undifferentiated schizophrenia and was prescribed antipsychotic medication. However, trials of risperidone, ziprasidone, aripiprazole, and quetiapine were each aborted within 10-15 days of commencement because her WBC count dropped into the 1600-2500/mm^3^ range. After 6 months of unsuccessful management without medications, she was started on lithium (300 mg/day) in the hope that this drug would stimulate leucocytosis and permit successful antipsychotic initiation.

Her pre-lithium WBC count was 2900/mm^3^; this rose to 3700/mm^3^ after two weeks of lithium therapy, at which point TFP were added. The TFP dose was 5 mg on the first day and 10 mg/day thereafter. By the end of a month of lithium-TFP combination therapy, psychosis was markedly attenuated. Treatment was continued for a further month and was then tapered and withdrawn at the insistence of the patient because she did not wish to continue experiencing the discomfort associated with the hematological monitoring. The WBC count remained stable within the range of 3400-3700/mm^3^ during the entire period of combination therapy.

She experienced a relapse of psychosis during 2008 and was successfully and uneventfully treated in a similar manner: she received TFP (10 mg/day) for one month after previous priming for 2 weeks with lithium (300 mg/day).

### Discussion

GCSF and lithium have both been used to treat blood dyscrasias that arise during antipsychotic therapy. Our case, however, is unique because there is no literature on the treatment of psychosis in patients with preexisting idiopathic blood dyscrasias that are worsened by antipsychotic drugs. The report nearest in nature to our case is that of Boshes *et al*.[10] These authors described a young male with preexisting leucopenia in whom lithium (600 mg/day; 0.63 mEq/L) was used not prophylactically but therapeutically for clozapine-induced worsening of leucopenia.

We accept that TFP may have had no adverse effect on WBC levels. However, considering that four previous antipsychotic drugs had each worsened the dyscrasia, we suggest an alternate explanation: that that even low-dose (300 mg/day) lithium pretreatment may help some patients with idiopathic blood dyscrasias (that are sensitive to psychotropic drugs) to successfully receive psychotropic medication. The successful use of lithium (0.6-1.1 mEq/L) to treat or prevent clozapine-induced neutropenia supports this view.

## DISCUSSION

We do not suggest that the final version that we present is the best possible version of the report. For example, more details could have been made available than have been presented. More references could have been cited than have been done. Perhaps references with greater contextual salience could have been identified. What we do assert, however, is that the final version that we present is one that most reviewers would find satisfactory.

Writing a good research paper is an art that requires skills in both academic and literary domains. We hope that readers who attempted the exercise advocated in this article will better understand how authors should prepare a report, and how they may anticipate concerns that peer reviewers may express when scrutinizing their manuscript. Manuscripts that are better in quality at the time of original submission will stand a higher chance of acceptance and will tax reviewers and editorial office staff less.
